# Individual differences in action co-representation: not personal distress or subclinical psychotic experiences but sex composition modulates joint action performance

**DOI:** 10.1007/s00221-015-4475-6

**Published:** 2015-11-02

**Authors:** Anouk van der Weiden, Henk Aarts, Merel Prikken, Neeltje E. M. van Haren

**Affiliations:** Brain Center Rudolf Magnus, University Medical Center Utrecht, Huispostnummer A.01.126, PO Box 85500, 3508 GA Utrecht, The Netherlands; Department of Psychology, Utrecht University, Heidelberglaan 1, 3584 CS Utrecht, The Netherlands

**Keywords:** Joint action, Simon effect, Self–other distinction, Subclinical psychotic symptoms, Personal distress, Sex composition

## Abstract

Successful social interaction requires the ability to integrate as well as distinguish own and others’ actions. Normally, the integration and distinction of self and other are a well-balanced process, occurring without much effort or conscious attention. However, not everyone is blessed with the ability to balance self–other distinction and integration, resulting in personal distress in reaction to other people’s emotions or even a loss of self [e.g., in (subclinical) psychosis]. Previous research has demonstrated that the integration and distinction of others’ actions cause interference with one’s own action performance (commonly assessed with a social Simon task). The present study had two goals. First, as previous studies on the social Simon effect employed relatively small samples (*N* < 50 per test), we aimed for a sample size that allowed us to test the robustness of the action interference effect. Second, we tested to what extent action interference reflects individual differences in traits related to self–other distinction (i.e., personal distress in reaction to other people’s emotions and subclinical psychotic symptoms). Based on a questionnaire study among a large sample (*N* = 745), we selected a subsample (*N* = 130) of participants scoring low, average, or high on subclinical psychotic symptoms, or on personal distress. The selected participants performed a social Simon task. Results showed a robust social Simon effect, regardless of individual differences in personal distress or subclinical psychotic symptoms. However, exploratory analyses revealed that the sex composition of interaction pairs modulated social Simon effects. Possible explanations for these findings are discussed.

## Introduction

As social beings, we develop great expertise in reading other people’s intentions, actions, and emotions. This ability has been linked to the so-called mirror neuron network, which is dedicated to understanding and anticipating *own* as well as *others’* actions and emotions (Cattaneo and Rizzolatti 2009; Gallese and Goldman 1998; Goldman 1989, 2009; Kilner et al. 2007; Meyer et al. 2011; Mukamel et al. 2010; Ocampo and Kritikos 2011). It allows people to take into account, or *integrate*, the actions and emotions of others and to react accordingly (e.g., to pass someone on the right when that person is going left, or to offer consolation when someone is feeling sad). Crucially, this neural network that allows self–other integration also allows people to *distinguish* own and others’ actions and emotions, as it is generally more active for own compared with others’ actions and emotions (Mukamel et al. 2010). In the present study, using a well-documented complementary action task that taps into these self–other integration and distinction processes (Dolk et al. 2013; Sartori and Betti 2015; Sebanz et al. 2003), we investigate the robustness of self–other distinction in social interaction, as well as its susceptibility to individual differences in traits related to self–other distinction.

The extent to which people integrate and distinguish self and other in social interaction has been extensively examined by using the joint (or social) Simon task. In a *typical* Simon task (Craft and Simon 1970), participants respond with left and right key presses to stimuli (e.g., red and green dots) that are presented to the left or to the right of the computer screen. This task requires people to distinguish left and right actions in terms of action planning and execution. As a consequence of this “left” versus “right” distinction, participants generally respond slower to stimuli that are spatially incongruent (e.g., pressing a left key in response to stimuli presented to the right). This interference effect is typically absent in a go/no-go version of the task where participants only have to respond to one of the stimuli (Hommel 1996; Sebanz et al. 2003). Intriguingly, when two participants each respond to one of the stimuli in a joint go/no-go Simon task, this reinstates the action interference effect. This reinstatement of the action interference effect is also known as the *social Simon* effect and reflects the extent to which one represents own actions in spatial reference to one’s co-actor (e.g., as “left” as opposed to “right”), resulting in slower reaction times to stimuli that are spatially incongruent with this representation (e.g., presented to the right; Dolk et al. 2013, 2014; Ferraro et al. 2011; Obhi and Sebanz 2011; Sebanz et al. 2003). Recent research shows that people may also use other (nonspatial) reference frames, such as color or identity (Philipp and Prinz 2010; Sellaro et al. 2015).

The social Simon effect varies in strength, partly due to the relatively small samples (usually around 20, with the highest *N* being 48 to our knowledge; Stenzel et al. 2013) that have been used in earlier work (Dolk et al. 2011; Ruys and Aarts 2010; Sebanz et al. 2003; Vlainic et al. 2010). Another reason for the variation in the strength of the social Simon effect pertains to the observation that the effect depends on contextual as well as individual differences (Aron et al. 1992; Colzato et al. 2012a, b, 2013; Decety and Sommerville 2003; Humphreys and Bedford 2011; Müller et al. 2011; Slotter and Gardner 2009). That is, although the social Simon effect is not necessarily a social effect (Dolk et al. 2013), it does depend on the social context. Specifically, the effect is stronger for similar versus dissimilar others, e.g., in terms of cognitive style (McClung et al. 2013), ethnicity (Müller et al. 2011) or perceived agency (Müller et al. 2011; Stenzel et al. 2012, 2014). Similarly, a focus on similarities or integration enhances the effect. For example, previous research suggests that convergent versus divergent thinking increases the social Simon effect (Colzato et al. 2013). Also, Buddhists who tend to integrate others more than atheists show a larger social Simon effect (Colzato et al. 2012). Thus, the social Simon effect may be regarded as an objective measure of self–other integration.

Although higher levels of self–other integration may help people cope better with threats (Castano et al. 2002) and increase compassion (Valdesolo and DeSteno 2011) and cooperation (Vesper et al. 2011; Wiltermuth and Heath, 2009), it may also be disturbing. For example, the simulation of (especially negative) emotions of others may result in personal distress, as can also be witnessed in occurrences of mass panic (Decety and Lamm 2011). Also, too much self–other integration may blur self–other boundaries and lead to a feeling of loss of self, which is a core characteristic of schizophrenia (Hur et al. 2014; Johns et al. 2001; Maeda et al. 2012; Mishara et al. 2014; Nelson et al. 2014; Renes et al. 2015; van der Weiden et al. 2015), and is also present in individuals with subclinical positive psychotic symptoms (Asai et al. 2011, 2008; Asai and Tanno 2008). Specifically, many (subclinical) psychotic symptoms (e.g., delusions of control, auditory hallucinations, grandiose delusions, and delusions of reference) reflect difficulties in distinguishing one’s own thoughts, emotions, intentions, and actions from those of others. For example, people may feel their actions are being controlled by others (i.e., delusions of control; Frith 2005; Stefanis et al. 2002).

To assess the extent to which experiences of personal distress in reaction to other people’s emotions and (subclinical) psychotic experiences reflect excessive integration of self and other, we aimed to test (1) the robustness of the social Simon effect, and (2) whether individual differences in personal distress and subclinical psychotic symptoms are reflected in enhanced social Simon effects. We expect that individuals who score high on personal distress or subclinical psychotic experiences integrate the other person’s actions too much (and distinguish too little), resulting in larger action interference, compared to those who score low or average on these traits. Conversely, people who score low on personal distress or subclinical psychotic symptoms may also show a weaker action interference effect than people who score average on these traits.

## Methods

### Ethics statement

This study has been approved by the local ethics committee and has therefore been performed in accordance with the ethical standards laid down in the 1964 Declaration of Helsinki. All participants gave their informed consent prior to their inclusion in the study.

### Participants and design

#### Participant recruitment

We administered two questionnaires in a large sample of young adults (*N* = 745; *M*_age_ = 20.88, SD_age_ = 2.37) to recruit participants scoring low versus high on personal distress and subclinical psychotic symptoms. For this purpose, we first administered the personal distress subscale as part of the Interpersonal Reactivity Index (IRI; Davis 1980, 1983), which assesses personal distress in reaction to other people’s emotions. This subscale consists of seven items that can be rated on a 5-point scale, ranging from 0 [*does not describe me well*] to 4 [*describes me very well*]. Example items are: “I sometimes feel helpless when I am in the middle of a very emotional situation” and “When I see someone who badly needs help in an emergency, I go to pieces.” The other subscales concern perspective-taking ability (perspective-taking subscale) and the ability to empathize with other people (empathy subscale) as well as fictive characters (fantasy subscale). Secondly, the severity of subclinical psychotic symptoms was assessed using the positive subscale of the Community Assessment of Psychic Experiences (CAPE; Stefanis et al. 2002). The positive subscale consists of 20 items that can be rated on a 4-point scale, ranging from 1 [*never*] to 4 [*almost constant*]. Example items are: “Have you ever felt as if the thoughts in your head are not your own?” and “Have you ever felt as if you are under the control of some force or power other than yourself?” The other subscales assess negative symptoms (i.e., absence of normal thoughts, feelings, and behaviors, e.g., affective flattening, apathy, anhedonia, and avolition) and symptoms of depression. Based on participants’ personal distress and positive psychotic symptom scores, we selected a subsample of participants (*N* = 130; *M*_age_ = 20.68, SD_age_ = 2.43) who scored low (lower than 1 SD from the total sample mean), average (equals total sample mean), or high (higher than 1 SD from the total sample mean) on either of these traits. All selected participants had normal or corrected-to-normal vision and were naïve to the purpose of this study.^1^ The large sample size (2.7 times larger than the largest sample size of 48) of our study allows us to test the robustness of the social Simon effect.

#### Total sample

**Fig. 1:**
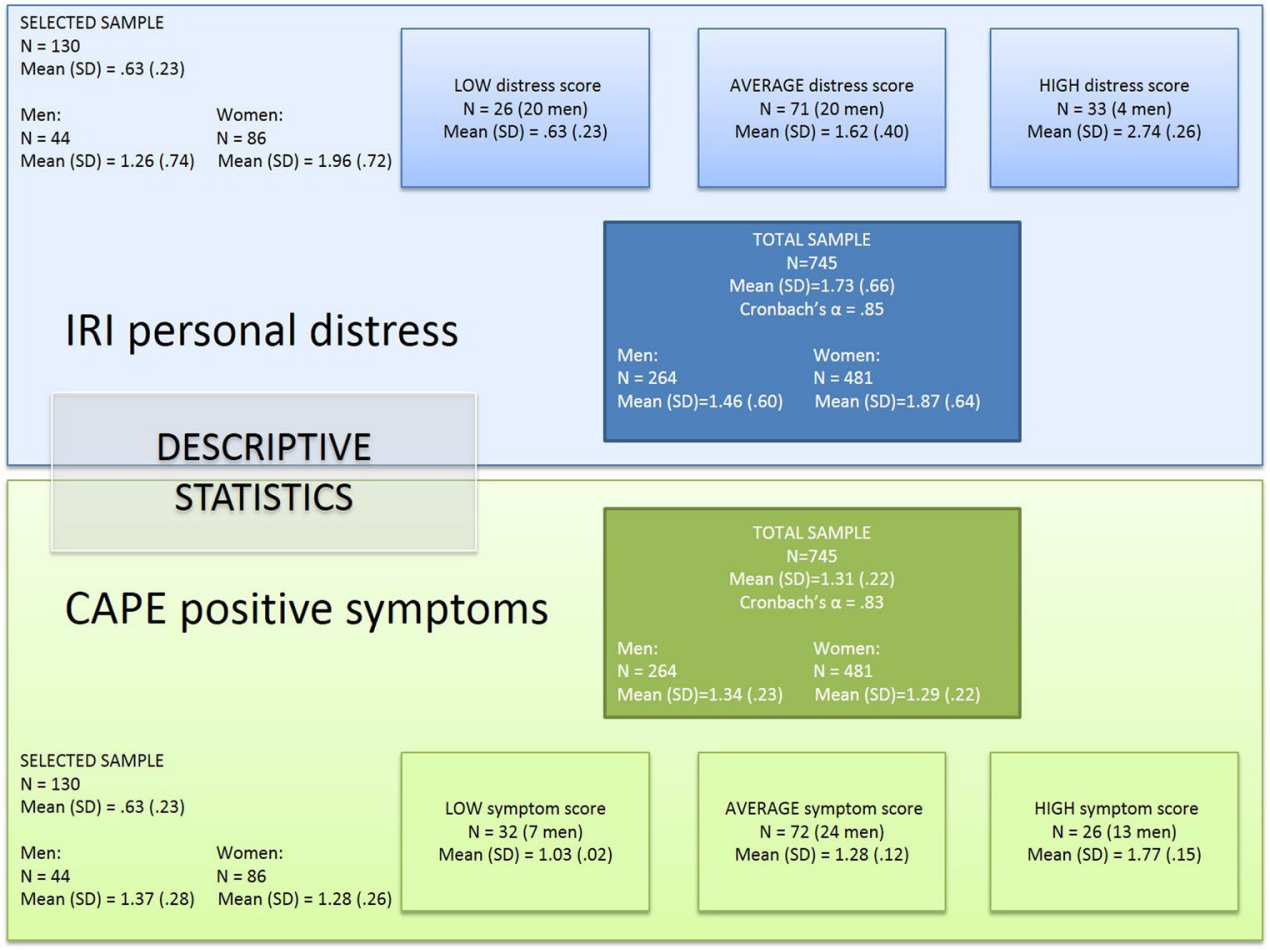
Descriptive statistics for the two subscales for the total and subsample

Figure 1 presents the descriptive statistics for the total sample. In line with previous research (Davis 1980; Michalska et al. 2013), women scored on average .41 higher on personal distress than men, with a 95 % CI of (.32, .51), *t*(742) = 8.57, *p* < .001, Cohen’s *d* = .66. Furthermore, men scored on average .05 higher on positive symptoms than women, with a 95 % CI of (.02, .09), *t*(740) = 3.06, *p* = .002, Cohen’s *d* = .24.

### Selected subsample

As participants filled in both questionnaires, they could end up in two of these groups (e.g., low on both traits; or low on one and high on the other trait). Importantly, the scores on the two subscales were not correlated, *r*(130) = −.08, *p* = .34. However, most participants who fell within one of the extreme groups (either high or low) of one trait had an average score on the other trait. Hence, the average groups were larger in sample size. See Fig. 1 for the descriptive statistics. The distributions of the selected samples did not differ from the total sample (no difference between selected and unselected participants as indicated by independent samples Mann–Whitney *U* tests: *p* = .87 for IRI_distress_ and *p* = .41 for CAPE_positive_). We therefore chose to analyze IRI and CAPE scores as continuous predictors.

Even more pronounced than in the whole sample (*F*(3,740) = 7.39, *p* = .01, *η*_*p*_^2^ = .01), women scored on average .70 higher on personal distress than men, with a 95 % CI of (.43, .97), *t*(127) = 5.15, *p* < .001, Cohen’s *d* = .97. Sex differences in psychotic symptoms were less pronounced in the selected sample, with men scoring only .09 higher on average than women, with a 95 % CI of (−.01, .19), *t*(127) = 1.76, *p* = .08, Cohen’s *d* = .33. Consequently, there were only a small number of women scoring low on IRI_distress_ and only a small number of men scoring high on IRI_distress_ in our selected sample. Similarly, there were only a small number of men scoring low on CAPE_positive_ in our selected sample.

We performed separate analyses for personal distress and subclinical psychotic symptoms. The experiment thus had a 1 (trait: CAPE_positive_ or IRI_distress_) by 2 (congruency: congruent vs. incongruent) mixed design, with trait as a continuous between-subjects variable and congruency as within-subjects variable. Because of sex differences on the IRI_distress_, we also included sex as a between-subjects variable in the analyses involving IRI_distress_.

### Experimental task and procedure

Participants were invited to the laboratory in random pairs to perform the validated and well-documented social Simon task to measure joint action interference (Sebanz et al. 2003). Mixed sex pairs were possible. The only criterion was that participants did not know each other. In case one of the participants in a pair did not show up, a research confederate performed his or her part of the task. In the social Simon task, participants sat next to each other as they responded to colored dots (i.e., red or green) on the computer screen. Each person was responsible for responding to only one of the two colors. For example, if the dot is red, the participant on the left presses the left button, and when the dot is green, the participants on the right press the right button (or vice versa). The location of the displayed dot was varied (left or right on the computer screen) to be congruent or incongruent with the subject’s location and button press. That is, the participant sitting on the left responded by pressing the “*Z*” on the left side of a QWERTY keyboard, and the participants sitting on the right responded by pressing “3” on the right numerical side of the same keyboard.

**Fig. 2:**
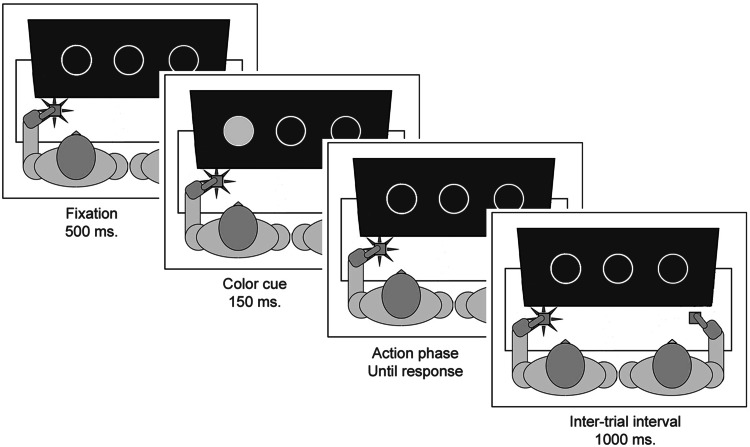
Schematic example of a congruent action trial in the social Simon task where the person on the left has to respond to *green* dots

At the beginning of each trial, participants saw a rectangular frame containing three circles (see Fig. 2 for an illustration of a trial). After 500 ms, one of these circles would turn either red or green for 150 ms. Depending on the color, either the participant on the left or the participant on the right had to respond as fast as possible, whereas the other participant had to refrain from responding. Once the participant had responded, the next trial would begin after a 1000-ms inter-trial interval.

The task consisted of 2 blocks of 45 trials each. In 20 of these trials, the location of the presented colored dot (e.g., green, left) was consistent with participants’ location and button press (i.e., left). In another 20 trials, the location of the colored dot (e.g., right) was inconsistent with participants’ location and button press (i.e., left). The remaining five trials were filler trials, in which the colored dot was presented in the middle. Trials were randomly presented. After the first block, the target color to which participants had to respond was counterbalanced. That is, participants who were responding to the color green in the first block had to respond to the color red in the second block, and vice versa.

## Results

Responses below 100 ms or above 1000 ms were removed (Ratcliff 1993). As color and seating location did not interact with congruency (all *F*s < 1.96), IRI_distress_ (all *F*s < 1.96), CAPE_pos_ (all *F*s < 2.66), or participants’ sex (all *F*s < 1.08) in any of the analyses reported below, we computed mean RTs (in ms) on congruent and incongruent trials, collapsing across target color and seating location. In 32 cases, a confederate played the part of co-actor. Importantly, this did not interact with the congruency effects reported below (all *F*s < 2.16). We subjected participants’ mean RTs to two separate 2 (congruency: incongruent vs. congruent) by 1 (standardized continuous trait score) repeated measures ANOVAs: one for personal distress and one for subclinical psychotic symptoms. Because of sex differences in self-reported personal distress, we further included participants’ sex as a between-participants factor in the personal distress analyses.

### Confirmatory analyses

#### Personal distress

**Fig. 3:**
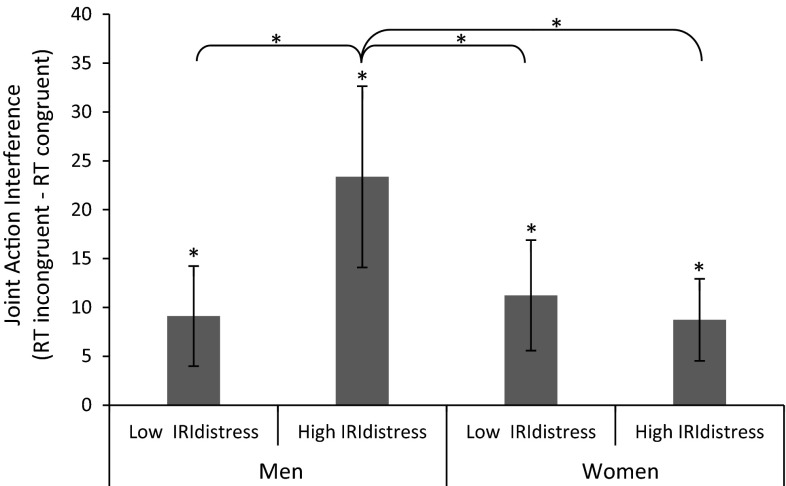
Joint action interference as a function of participants’ sex and individual differences in personal distress in reaction to other people’s emotions. *Error bars* represent 95 % confidence intervals

A repeated measures ANOVA with congruency (incongruent vs. congruent) as within-subjects variable, IRI_distress_ as standardized continuous predictor, and participants’ sex (male vs. female) as between-subjects variable revealed the expected main effect of congruency. Participants were on average 10.07 ms faster to respond to congruent (*M* = 314.12, SD = 35.65) compared with incongruent (*M* = 324.19, SD = 36.18) stimuli. As expected, there was an interaction between congruency and IRI_distress_ (see upper right cell in Table 1). However, this interaction was qualified by an unexpected three-way interaction with participants’ sex (see lower right cell in Table 1). Figure 3 presents the mean RTs for each cell in the design; Table 1 presents the statistics.

**Table 1:** Statistical analyses for congruency, IRI_distress_, and participants’ sex

Error *df* = 126	Total sample			Low IRI_distress_			High IRI_distress_			Low versus high IRI_distress_		
	*F*	Sig.	*η*_*p*_^2^	*F*	Sig.	*η*_*p*_^2^	*F*	Sig.	*η*_*p*_^2^	*F*	Sig.	*η*_*p*_^2^
Congruency	54.38	<.001	.30	27.87	<.001	.18	38.94	<.001	.24	4.30	.04	.03
Men	41.90	<.001	.50^a^	12.40	.001	.09	24.89	<.001	.17	14.11	.001	.25^b^
Women	24.43	<.001	.23^a^	15.47	<.001	.11	16.88	<.001	.12	.49	.49	.01^c^
Men versus women	3.10	.08	.02	.30	.58	.002	8.10	.005	.06	8.76	.004	.07

^a^ *df* = 128

^b^ *df* = 42

^c^ *df* = 84

In order to examine this three-way interaction and to test our specific hypothesis, the effect of congruency was assessed for male and female participants scoring low on IRI_distress_ (one standard deviation below the mean) and for male and female participants scoring high on IRI_distress_ (one standard deviation above the mean) separately (based on estimated marginal means; see Aiken and West 1991). These analyses revealed that male participants scoring high on IRI_distress_ had a larger congruency effect than male participants scoring low on IRI_distress_ and female participants in general. However, considering that only a small proportion of males actually scored high (>1 SD above the mean) on personal distress, these effects should be interpreted with extreme caution.

#### Subclinical psychotic symptoms

**Fig. 4:**
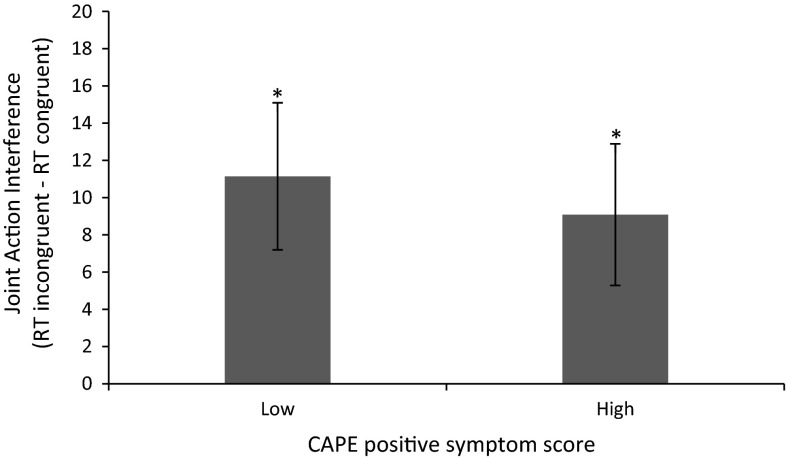
Joint action interference as a function of participants’ sex and individual differences in subclinical psychotic symptoms (based on estimated marginal means). *Error bars* represent 95 % confidence intervals

A repeated measures ANOVA with congruency (incongruent vs. congruent) as within-subjects variable and CAPE_positive_ as standardized continuous predictor again revealed the main effect of congruency. Contrary to our expectations, there were no main or interaction effects with CAPE_positive_. Figure 4 presents the mean RTs for each cell in the design; Table 2 presents the statistics.

**Table 2:** Statistical analyses for congruency, CAPE_positive_, and participants’ sex

Error *df* = 128	*F*	Sig.	*η*_*p*_^2^
Congruency	44.96	<.001	.26
CAPE_positive_	1.63	.20	.01
Congruency * CAPE_positive_	.68	.41	.01

### Exploratory post hoc analyses

#### Social Simon effect and the other IRI and CAPE subscales

As the distribution of the personal distress and positive psychotic symptom scales in the selected sample were normally distributed and did not differ from the total sample, we were also able to explore potential relationships between the other subscales and the social Simon effect. In particular, the perspective-taking subscale of the IRI is potentially interesting as recent research has shown that people show a stronger social Simon effect when they take the perspective of their interaction partner (Ford and Aberdein 2015; Müller et al. 2011a, b, 2015).

To assess the relationship between the social Simon effect and the other subscales, we performed a number of separate repeated measures ANOVAs with congruency (incongruent vs. congruent) as within-subjects variable, and the different subscales as standardized continuous variable. As women scored higher on perspective taking (*t*(128) = 2.44, *p* = .02), empathy (*t*(128) = 6.82, *p* < .001), and fantasy (*t*(128) = 2.79, *p* = .01), participant’s sex (male vs. female) was also included as between-subjects variable in the analyses concerning these subscales. These analyses again revealed strong main effects of congruency (all *F*s ≥ 30.88), but no interactions between congruency and any of the other subscales (all *F*s ≤ 1.75).

#### Sex composition

In our study, we included both male and female participants. It has been argued that participants’ sex may affect the joint action effect because of in-group/out-group categorization processes (Powlishta 1995). For this purpose, some researchers took sex into account by studying the social Simon effect in matched gender pairs or even including only male or female participants in their studies (Liepelt et al. 2012; McClung et al. 2013; Philipp and Prinz 2010). As far as we know, there is only one study that showed effects of sex composition on the social Simon effect, such that the social Simon effect is stronger in same-sex pairs than in opposite-sex pairs (Mussi et al. 2015). Furthermore, sample sizes are usually too small to analyze the effect of sex composition and potential sex differences herein. Because of our relatively large sample size, we are able to provide a first test of sex composition in the joint action effect. In doing so, we controlled for individual differences in IRI_distress_, as men and women differed on this trait.

##### Action interference as a function of own and other’s sex

A repeated measures ANOVA with congruency (incongruent vs. congruent) as within-subjects variable, participant’s sex (male vs. female) and partner’s sex (male vs. female) as between-subjects variables, and IRI_distress_ as standardized continuous variable again revealed a strong main effect of congruency, as well as an interaction between congruency and IRI_distress_, and a three-way interaction with participants’ sex. Additionally, the analysis revealed an interaction between congruency and participants’ sex, which was qualified by a reliable three-way interaction of congruency, participant’s sex, and partners’ sex. See Table 3 for the statistics.

**Table 3:** Statistical analyses for congruency, participants’ sex, partners’ sex, and IRI_distress_

Error *df* = 128	Opposite-sex pairs			Same-sex pairs			Opposite- versus same-sex pairs		
	*F*	Sig.	*η*_*p*_^2^	*F*	Sig.	*η*_*p*_^2^	*F*	Sig.	*η*_*p*_^2^
Congruency	6.35	.01	.05	44.67	<.001	.26	4.94	.03	.04

Error *df* = 122	Male interaction partner			Female interaction partner			Male versus female interaction partner		
	*F*	Sig.	*η*_*p*_^2^	*F*	Sig.	*η*_*p*_^2^	*F*	Sig.	*η*_*p*_^2^
Participant’s sex	.01	.93	<.001^a^	1.52	.22	.01^a^	3.16	.08	.03
Congruency	11.64	.001	.08	35.86	<.001	.22	.82	.37	.01
Men	13.36	<.001	.10	5.85	.02	.04	.65	.42	.01
Women	1.88	.17	.01	30.79	<.001	.20	4.86	.03	.04
Men versus women	4.01	.05	.03	1.35	.25	.01	5.15	.03	.04

Error *df* = 122	Total sample			Low IRI_distress_			High IRI_distress_			Low versus high IRI_distress_		
	*F*	Sig.	*η*_*p*_^2^	*F*	Sig.	*η*_*p*_^2^	*F*	Sig.	*η*_*p*_^2^	*F*	Sig.	*η*_*p*_^2^
Congruency	53.58	<.001	.31	26.46	<.001	.18	39.04	<.001	.24	4.94	.03	.04
Men	18.61	<.001	.13	12.78	.001	.10	26.34	<.001	.18	14.06	<.001	.26^a^
Women	27.40	<.001	.18	13.71	<.001	.10	13.98	<.001	.10	.33	.57	.004^b^
Men versus women	3.94	.05	.03	.12	.73	.001	8.93	.003	.07	8.74	.004	.07

^a^ *df* = 125

^b^ *df* = 126

To gain further insight into this three-way interaction, we performed simple effects analyses. To corroborate the recent findings of Mussi et al. (2015), we first analyzed the social Simon effect as a function of same-sex versus opposite-sex pairing. These analyses revealed that although there was an effect of congruency for both same-sex and opposite-sex pairs, the congruency effect was larger by 6.75 ms for same-sex pairs (*M* = 12.71, SD = 17.23) compared with opposite-sex pairs (*M* = 5.97, SD = 16.39), 95 % CI (.74, 12.76). To further inspect this two-way interaction, we performed further simple effects analyses to investigate the effect of congruency and sex composition within women and men separately.

For women, a reliable two-way interaction emerged between partners’ sex and congruency. On average, women responded 12.81 ms faster to congruent (*M* = 313.04, SD = 33.68) compared with incongruent (*M* = 324.89, SD = 33.99) stimuli with a 95 % CI of (7.24, 16.46) when they interacted with another woman. When they interacted with a man, RTs did not differ for congruent (*M* = 314.40, SD = 37.76) and incongruent (*M* = 319.00, SD = 34.49) stimuli, 95 % CI (−2.34, 11.54).

**Fig. 5:**
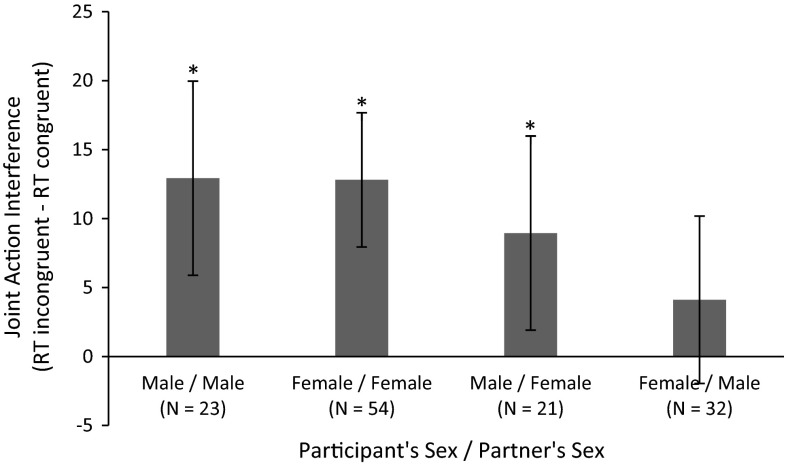
Joint action interference for the different compositions of pairs in terms of participant’s sex and interaction partner’s sex. Note that the number of men performing the task with a female co-actor differs from the number of women performing the task with a male co-actor. This is due to the occasional interaction with a research confederate rather than another participant as a co-actor. *Error bars* represent 95 % confidence intervals

For men, there was no statistically significant interaction between partners’ sex and congruency. Men responded 11.03 ms faster on average to congruent (*M* = 315.39, SD = 37.66) compared with incongruent (*M* = 326.42, SD = 40.33) stimuli [95 % CI (6.21, 15.85)], regardless of their interaction partner’s sex. Figure 5 presents the mean RTs for each cell in the design.

##### Reaction times as a function of own and other’s sex

**Fig. 6:**
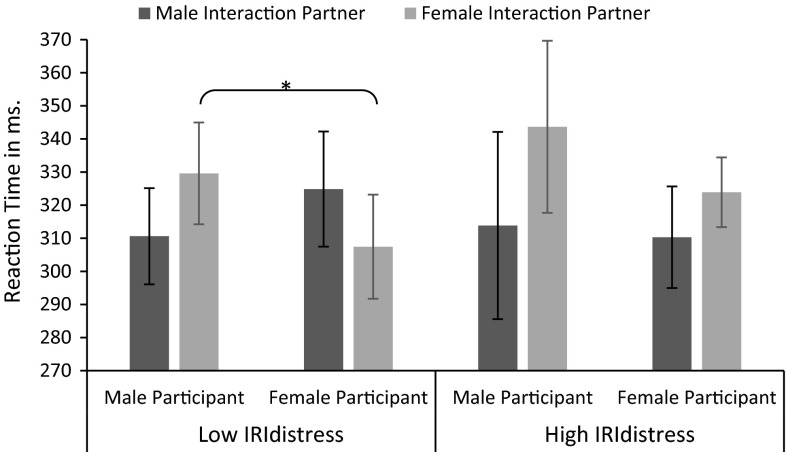
Mean reaction times as a function of IRI_distress_ score, participant’s sex, and interaction partner’s sex. *Error bars* represent 95 % confidence intervals

The repeated measures ANOVA further revealed a statistically nonsignificant interaction effect between participants’ sex and partners’ sex. Specifically, male participants interacting with a female partner seem to perform much slower (*M* = 331.78, SD = 45.40) compared with male participants interacting with a male partner (*M* = 310.99, SD = 29.63) and female participants in general (*M* = 317.91, SD = 34.42). This interaction seems to mainly be driven by a decrease in reaction time for men interacting with women and is slightly stronger for participants scoring low on IRI_distress_ than for participants scoring high on IRI_distress_. Figure 6 presents the mean reaction times as a function of participants’ sex, partners’ sex, and IRI_distress_.

## Discussion and conclusion

The present study examined the effect of individual differences in level of experienced personal distress and subclinical psychotic symptoms on action interference in joint action. As expected, we replicated the basic social Simon effect in a large sample (*N* = 130), speaking for the robustness of the effect. In contrast to our hypotheses, individual differences in subclinical psychotic symptoms and interpersonal distress did not seem to reliably affect action interference effects in joint action. Although there was a statistically significant interaction between interpersonal distress and joint action interference, this interaction was only driven by a small subsample of male participants and should thus be interpreted with caution. Finally, exploratory analyses revealed that action interference depended on the gender composition of interaction pairs.

### Personal distress

The present findings seem to suggest that although people who tend to experience distress in reaction to other people’s emotions may have difficulty in distinguishing own and others’ *emotions* (Lamm et al. 2007b), they do not necessarily struggle with distinguishing own and others’ *actions* (see also Ford and Aberdein 2015). This may be because others’ emotions are typically more salient than the neutral and irrelevant actions of the interaction partner in our experimental setup. If so, increasing the salience of the interaction partners’ actions may result in stronger action interference. In line with this notion, recent research has indicated that action interference reduces as the action event becomes less social and less salient (Dolk et al. 2013; Klempova and Liepelt 2015).

Furthermore, the integration of own and others’ *actions* involves different brain areas than the integration of own and others’ *emotions* (Carr et al. 2003; Cochin et al. 1998; Hari et al. 1998; Iacoboni 2009; Mukamel et al. 2010; Rizzolatti and Craighero 2005). That is, while the observation of others’ actions and emotions activates overlapping brain areas in the mirror neuron network, the observation of others’ emotions additionally activates areas in the limbic system (Carr et al. 2003; Hefner et al. 2008; Lamm et al. 2007a; Muigg et al. 2008). Although the limbic system is involved in the understanding and performance of *emotion*-*laden* actions (e.g., scoring a goal in a soccer game; Hajcak et al. 2007; Mogenson et al. 1980), it may not be involved in the understanding and performance of *neutral* actions (e.g., pressing a left button in response to a green dot). Hence, variability in experiencing distress in reaction to other people’s emotions may be related to specific processes in the limbic system and may only affect the coordination of emotion-laden actions.

### Subclinical psychotic symptoms

People who scored relatively high on subclinical psychotic symptoms also did not seem to have difficulty distinguishing own and others’ actions. Whereas previous work has found relations between subclinical psychotic symptoms and the sense of agency over action (Asai and Tanno 2008; Hauser et al. 2011; Sugimori et al. 2011; but see Jones et al. 2008), the present findings indicate that these relations cannot be generalized to self–other distinction at the behavioral level. However, it is important to note that even in the “high” subclinical psychotic symptoms group, psychotic experiences were still infrequent (1.77 on average with 1 being “never”). This is in line with the notion that although subclinical positive symptoms are highly prevalent in the general population (around 100 times greater than clinical psychosis), these symptoms are usually not persistent (Hanssen et al. 2005). The chance of observing a relation between subclinical positive symptoms and joint action performance might thus be bigger if they would be assessed over a shorter time span.

Another direction for future research is to further examine joint action performance in individuals with a more stable and/or severe condition, such as in people with schizotypal personality traits or in patients with schizophrenia. So far, only one study has been conducted that examined the action interference effect in schizophrenia patients (Liepelt et al. 2012). Results indicated that schizophrenia patients do not experience any interference from their interaction partner’s actions. This lack of action interference may reflect a lack of co-representing (or *integrating)* the partners’ actions, or alternatively an inability to *distinguish* between own and others’ actions based on spatial cues (Dolk et al. 2013). Either of these impairments would undermine successful action coordination. For example, a lack of integration might actually facilitate one’s own action performance (e.g., throwing a ball), but would impede joint action performance (e.g., throwing a ball in such a way that one’s friend can catch it and throw it back). However, a lack of distinction would impede one’s own action performance as well as joint action performance (e.g., making a throwing movement when one should catch the ball). Hence, it would be interesting for future research to examine whether the absence of action interference in schizophrenia patients results from a lack of integration of and/or distinction between own and others’ actions.

### Other IRI and CAPE subscales

Although previous research has demonstrated that the social Simon effect is generally stronger when people take the perspective of their interaction partner (Müller et al. 2011a, b, 2015), we were unable to pick up this effect when using a self-report questionnaire that assesses people’s tendency to take the perspective of others in daily life. This converges with recent evidence that the social Simon effect is only modulated by self-reported empathy when interacting with a friend, and not when interacting with a stranger (Ford and Aberdein 2015). These findings suggest that perspective-taking ability is not sufficient to enhance the social Simon effect. That is, whether this ability is actually deployed also depends on the context, e.g., whether one’s interaction partner is a friend (Ford and Aberdein 2015), or whether one is explicitly instructed to take the perspective of one’s interaction partner (Müller et al. 2011a, b, 2015). Similarly, the social Simon effect may be more susceptible to state manipulations of empathy, fantasy, or personal distress than individual trait differences herein.

### Sex composition

Replicating and extending recent work (Mussi et al. 2015), our exploratory analyses indicated that the sex composition of interaction pairs modulated action interference effects. First of all, although this effect has to be interpreted with caution, there was a trend for men to be overall faster when interacting with men compared with women. This may be either because men tend to get distracted by women (Duncan et al. 2007; van Hooff et al. 2011; Zhang and Deng 2014), or because men are more competitive when interacting with another man (Cashdan 1998; Freischlag 1973). These processes may also have played a role in the action interference effects. That is, men showed an action interference effect, regardless of their interaction partners’ sex. However, these action interference effects may have resulted from different processes, since action interference is enhanced by attention (Dittrich et al. 2012) as well as competition (Ruys et al. 2010). It is thus important to consider the sex of participants as well as their interaction partners if we want to better understand the underlying mechanisms of and implications for social interaction.

This is also reflected in the observation that women only experienced action interference when interacting with another woman, while they experienced no action interference when interacting with men. This suggests that women either do not integrate or very effectively distinguish the actions of unknown men, supporting the notion that sex may instigate in-group/out-group categorization processes (Powlishta 1995). In line with this notion, action interference effects in general were stronger for same-sex pairs compared with opposite-sex pairs. Importantly, as only women showed an absence of the action interference effect when interacting with an interaction partner of the opposite sex, there may be factors that modulate this effect, such as an actor’s sexual goals (Aarts et al. 2004; Karremans and Verwijmeren 2008; Petersen and Hyde 2010). For example, recent research suggests that people who are involved in a romantic relationship are less inclined to mimic the behaviors of opposite-sex others (Karremans and Verwijmeren 2008). If women in our sample were more often involved in romantic relationships than men, this may explain the specific absence of an action interference effect for women interacting with men. Furthermore, men may be more attentive to women because they more often pursue casual sex than women (Clark and Hatfield 1989; Ickes 1993; Leitenberg and Henning 1995). It would be interesting for future research to address the role of gender as well as relationship status in joint action interference.

In conclusion, the present study suggests that individual differences in personal distress and subclinical psychotic symptoms do not reliably affect the performance of neutral, complementary actions. However, extending recent research (Mussi et al. 2015), we provided a first, exploratory test of different sex compositions in joint action performance. Although the present findings have to be interpreted with caution and need to be replicated in future research, they suggest that it is important to consider the sex of participants as well as their interaction partners if we want to better understand the underlying mechanisms of and implications for social interaction.

## Footnotes

1 Unfortunately we did not determine handedness, but as participants were free to use their left or right hand for responding, and seating location did not affect our results, we believe it is not very likely that the dominance of handedness had a substantial influence on our results.
